# Four decades of socio-economic inequality and secular change in the physical growth of Guatemalans

**DOI:** 10.1017/S1368980019003239

**Published:** 2020-06

**Authors:** Liina Mansukoski, William Johnson, Katherine Brooke-Wavell, J Andres Galvez-Sobral, Luis Furlán, Tim J Cole, Barry Bogin

**Affiliations:** 1 School of Sport, Exercise and Health Sciences, Loughborough University, Loughborough, UK; 2 Centro de Investigaciones Educativas, Universidad del Valle de Guatemala, Guatemala City, Guatemala; 3 Centro de Estudios en Informática Aplicada, Universidad del Valle de Guatemala, Guatemala City, Guatemala; 4 Great Ormond Street Institute of Child Health, University College London, London, UK

**Keywords:** Super-Imposition by Translationand Rotation (SITAR), Pubertal timing, Growth inequalities, Dual burden

## Abstract

**Objective:**

To investigate changes in socio-economic inequalities in growth in height, weight, BMI and grip strength in children born during 1955–1993 in Guatemala, a period of marked socio-economic-political change.

**Design:**

We modelled longitudinal data on height, weight, BMI and hand grip strength using Super-Imposition by Translation and Rotation (SITAR). Internal *Z*-scores summarising growth size, timing and intensity (peak growth velocity, e.g. cm/year) were created to investigate inequalities by socio-economic position (SEP; measured by school attended). Interactions of SEP with date of birth were investigated to capture secular changes in inequalities.

**Setting:**

Urban and peri-urban schools in the region of Guatemala City, Guatemala.

**Participants:**

Participants were 40 484 children and adolescents aged 3–19 years of Ladino and Maya ancestry (*n*_observations_ 157 067).

**Results:**

The difference in height (SITAR size) between lowest and highest SEP decreased from −2·0 (95 % CI −2·2, −1·9) sd to −1·4 (95 % CI −1·5, −1·3) sd in males, and from −2·0 (95 % CI −2·1, −1·9) sd to −1·2 (95 % CI −1·3, −1·2) sd in females over the study period. Inequalities also reduced for weight, BMI and grip strength, due to greater secular increases in lowest-SEP groups. The puberty period was earlier and shorter in higher-SEP individuals (earlier SITAR timing and higher SITAR intensity). All SEP groups showed increases in BMI intensity over time.

**Conclusions:**

Inequality narrowed between the 1960s and 1990s. The lowest-SEP groups were still >1 sd shorter than the highest. Risks remain for reduced human capital and poorer population health for urban Guatemalans.

Growth is a powerful predictor of later-life health and disease risk^(1)^. Growth faltering in both height and weight in its most extreme form causes death, and in lesser forms, is linked to increased disease risk later in life, poorer economic productivity and lower educational attainment^(2,3)^. According to UN Sustainable Development Goals 2 and 3, it is a global health priority to understand inequalities in health and growth, and to target interventions accordingly^(4)^. In a broader biocultural sense, child height and weight are reflective of the general living conditions of a society, including access to appropriate nutrition, health care, education, emotional well-being and money^(5)^. Macroeconomic growth *per se* does not seem to reduce rates of growth faltering but there is a consistent relationship between socio-economic position (SEP) and growth within populations^(6,7)^. Recent work from the UK has shown that patterns of inequality in child height, weight and BMI change through time, reflecting changes in the overall living conditions^(7,8)^.

Many low- and middle-income countries are characterised by large socio-economic inequality (commonly measured by the Gini Index) and are therefore likely to show larger differences in physical growth between SEP groups compared with countries such as the UK. Guatemala is both one of the most income inequal and most stunted countries in the world^(9)^. The population experienced a devastating civil war between 1960 and 1996^(10)^. Despite the signing of peace accords in 1996, violence continues until this day. During the civil war the indigenous population of Guatemala, the Maya, suffered extreme violence, oppression, and lack of access to education and health care. Today, up to 70 % of children in highland Maya populations are stunted^(11)^. About half of the population of Guatemala is considered Maya, with the other half identifying as ‘Ladino’, descendants of predominantly Spanish, Spanish-Maya and other ethnic origins. Over the last 100 years, there has been a 9·6 cm increase in the average height of Guatemalan males and a 9·1 cm increase for Guatemalan females, but even so the women are the shortest of any living population and the men the 11th shortest^(2)^. Due to the alarming global rates of obesity, weight and body-composition-related growth measures are important to study alongside height. Guatemalan obesity rates are not formally monitored, but nationally representative data from the Guatemalan Living Standard Measurement Survey (ENCOVI 2000) estimated that between 1995 and 2000 the adult obesity rate increased from 8·1 to 16·0 %, while 7·2 % of urban Guatemalan children were estimated to be obese^(9)^. For females, the rates are the highest in Latin America. A continuation of this trend has been described by the most recent Guatemalan Demographic and Health Survey from 2015^(12)^.

Historically, comprehensive longitudinal growth and health data from countries outside Europe and North America are largely absent. Uniquely, in Guatemala there have been two large child growth and health studies: the Institute of Nutrition of Central America and Panama (INCAP) Study and the Universidad del Valle de Guatemala (UVG) Longitudinal Study of Child and Adolescent Development^(13–15)^. The INCAP Study introduced a nutritional intervention to resource-deprived rural, Ladino-only Guatemalan villages, and a follow-up in the late 1980s showed that the benefits of this intervention carried into adulthood in terms of economic and educational success^(16,17)^. The UVG Study was started in 1953 and continued until 1999 with over 40 000 schoolchildren measured annually at six urban Ladino schools in Guatemala City and a semi-urban Maya school 25 km from the City. Differences in physical growth, bone maturation and cognitive measures were reported between high-, middle- and low-SEP individuals, whereby the Maya in particular suffered from growth faltering in comparison to the other groups^(15,18,19)^. These analyses were based on relatively small sub-samples of the UVG Study and did not report on secular changes in growth. It is further unknown if there have been changes over time in the timing and intensity of physical growth. The aim of the present study was to investigate how SEP inequalities in growth pattern of height, weight, BMI and hand grip strength have changed for individuals born between 1955 and 1993 (a time of great socio-economic and political change) using the full UVG Study database.

## Methods

### Study design and participants

We carried out a retrospective cohort study using longitudinal growth data of children who participated in the UVG Longitudinal Study of Child and Adolescent Development. Each year, a team of investigators visited seven study schools and measured all the students in attendance. The time elapsed between measurements usually ranged between 0·95 and 1·05 years^(14)^. Some participants were seen only twice and others between three and thirteen times. The schools were chosen based on parental SEP as measured by the school fees and parental education and occupation^(15)^. Upon enrolment in a school the children were automatically entered into the study and informed consent for participation was collected from their parents.

### Procedures

Standard anthropometric measurements were taken by a trained research team from the UVG following the guidelines of the International Biological Program; detailed protocols have been described previously^(14,18)^. For the present study, we used the data collected for height (cm), weight (kg) and hand grip strength (kg) with participants seated. BMI (kg/m^2^) was calculated from height and weight. The 40 848 individuals in the study yielded a total of 157 067 mixed longitudinal observations, see Table 1 for summary statistics.

**Table 1:** Summary statistics: numbers of individuals (*n*) with mean and sd for age and anthropometric outcomes, for the sample of children and adolescents (*n* 40 484) aged 3–19 years of Ladino and Maya ancestry born during 1955–1993 in Guatemala (*n*_observations_ 157 067)

Variable	*n*	Mean	sd
Age (years)	40 484	11·0	3·3
Height (cm)	40 484	138·9	18·2
Weight (kg)	40 479	37·2	14·7
BMI (kg/m^2^)	40 478	18·4	3·2
Grip strength (kg)	40 411	18·8	10·7

### Ascertainment of socio-economic position

A SEP variable was created for the present study from the data of the school attended. Data are available from schools ranging from the most expensive private schools to underprivileged state schools, allowing investigations of population-level inequality^(14,15)^. To reiterate, schools were chosen for the original study based on the SEP of the students’ families, as measured by the parents’ education and occupation and fees paid to the schools^(14)^. For the very early years of the study, data were not available for all seven schools and therefore SEP analyses were restricted to individuals born after 1955 (*n* 38 672), measurements were taken between 1963 and 1999. The highest SEP group (SEP 1, *n* 6369) consisted of individuals who attended a private institution charging high fees. SEP 2 (*n* 15 849) was made up of students who attended single-sex private Catholic schools with moderate fees. SEP 3 (*n* 3279) consisted of students attending two co-educational Catholic private institutions with low fees or no fee. SEP 4 (*n* 8508) included the students of a co-educational, state-run, non-sectarian institution with no fee. The students from SEP 1 to SEP 4 were virtually all Ladinos. SEP 5 (*n* 4667) consisted of students of two co-educational, state-run, non-sectarian schools with no fee, attended primarily by individuals of Maya ethnicity. The school-based assessment of SEP in the original study was investigated with a separate assessment for four of the study schools when the study was still ongoing^(15)^. A composite SEP score (range 4–15), representing parental occupation, parental education and zone of residence in the City, supported the school-based SEP categorisation^(15)^. The analysis included a random sample of 672 families with children attending the study schools. The school classified here as SEP 1 had a mean composite score of 12·2 (sd 3·4) and the two schools grouped as SEP 2 had a score of 10·2 (sd 6·6)^(15)^. For the school categorised as SEP 4 the score was 5·75 (sd 0·4). Unfortunately, the schools that in the present study constitute SEP 3 and 5 were not assessed, and there are no records or data which could be used to retrospectively assess or assign SEP at the individual or family level beyond school attended by participants.

### Statistical analysis

In the first stage of analysis we used Super-Imposition by Translation and Rotation (SITAR). This is a data reduction technique which results in three biologically meaningful traits summarising growth for each child. SITAR growth models for childhood height, weight, BMI and grip strength were created using the entire longitudinal database from the UVG Study. The SITAR growth curve model fits a smooth mean curve to the data (as a natural cubic B-spline) and at the same time it fits random effects for individuals that consider differences in mean size, pubertal timing and pubertal intensity. The strength of this approach is that the random effects are biologically meaningful, yet simple, transformations of the mean curve that can be visualised geometrically. The three traits are:

1. ‘Size’, which indicates by how much the mean curve is shifted up or down to best match the individual’s curve, where larger size is positive and smaller negative, measured in units of the outcome (e.g. cm for height).
2. ‘Timing’, which relates to the age at peak growth velocity (APV) and indicates by how much the mean curve is shifted left or right to best match the APV on the individual’s curve, where positive timing indicates later maturation and negative earlier maturation, measured in years.
3. ‘Intensity’, which relates to the peak growth velocity (PV) and indicates by how much the mean curve is adjusted (see below) to best match the PV on the individual’s curve, where positive intensity indicates greater PV and negative smaller PV, measured as a fraction of the mean PV.

The key assumption of SITAR is that applying each individual’s three transformations to the mean curve makes it a close match to the individual’s own curve^(20)^. Equivalently, applying the inverse transformations to individual curves (i.e. with reversed sign) causes them to match the mean curve, so all the curves are superimposed. Size, timing and intensity are also estimated as fixed effects, so the random effects have a mean of 0. SITAR has been shown to provide an unbiased estimate of APV^(20)^. The SITAR formula is:

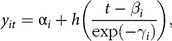

where *y*_*it*_ is the height of subject *i* at age *t*, *h*(*t*) is a natural cubic spline curve of height *v*. age and *α*_*i*_, *β*_*i*_ and *γ*_*i*_ are subject-specific random effects^(20)^.

To develop the most appropriate models, various transformations of the outcome and age were compared using the Bayesian information criterion. The model for height benefited from a log-age transformation and the models for weight and BMI from a log transformation of the outcome. The BMI and grip strength models fitted better without the timing fixed effect. All models were fitted to girls and boys separately, as the mean curves are known to differ by sex.

In the second stage of analysis, the individual random effects derived from the SITAR models were transformed to internal *Z*-scores, by sex, to allow for easier comparisons between outcomes with different units of measurement. Linear regression models were used to investigate variation in size, timing and intensity according to SEP group and date of birth in the R programming language and software environment for statistical computing^(21)^. Models were outcome (size, timing, intensity of each measure) and sex specific. The interaction between SEP group and date of birth was added to capture whether SEP inequality increased or narrowed through time. The unit of measurement for date of birth was changed by dividing the decimal dates of birth by 10 (unit changed from year to decade) and the data were centred on the mean of the date of birth distribution. This allowed for meaningful interpretation of effect sizes whereby each decade increase in date of birth is associated with *B* × *Z*-score increase in the outcome. The regression estimates were centred at the beginning of full decades within the date of birth range, meaning the years 1960, 1970, 1980 and 1990. These centred values estimate the SEP inequality at the different time points and were used to plot the figures. SEP 1 was assigned as the ‘healthy’ reference group because, compared with the current WHO growth standards, individuals from SEP 1 are close to the WHO mean with an average height of −0·2 WHO *Z*-scores across all ages (Table 2).

**Table 2:** Mean WHO* *Z*-scores and sd at age 5–19 years for height and BMI, prevalence of stunting (height-for-age *Z*-score ≤ −2) and prevalence of obesity (BMI-for-age *Z*-score ≥ +2) by socio-economic position (SEP) group in the sample of children and adolescents (*n* 40 484) aged 3–19 years of Ladino and Maya ancestry born during 1955–1993 in Guatemala (*n*_observations_ 157 067)

Variable	SEP 1	SEP 2	SEP 3	SEP 4	SEP 5
Mean WHO height-for-age *Z*-score	−0·2	−0·5	−1·3	−1·7	−2·0
sd	1·0	1·0	1·0	1·0	0·9
Prevalence of stunting across ages (%)	2·9	4·7	23·5	39·9	52·0
Mean WHO BMI-for-age *Z*-score	0·3	0·4	0·2	0·0	0·0
sd	1·0	1·1	1·0	0·9	0·8
Prevalence of obesity across ages (%)	5·1	9·3	4·3	2·1	1·1

* de Onis *et al*.^(46)^. Weight-for-age *Z*-scores were not calculated as the WHO standards for weight only include ages up to 10 years.

## Results

**Fig. 1:**
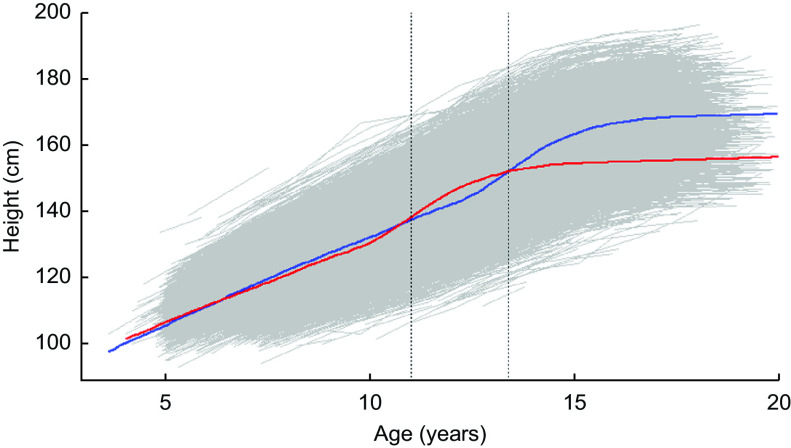
Super-Imposition by Translation and Rotation (SITAR) average male (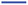) and female (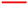) height (in centimetres) by age (in years) curve, with age at peak velocity (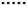; first vertical line for females, second vertical line for males) and with raw data (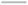) in the background, for children and adolescents (*n* 40 484) aged 3–19 years of Ladino and Maya ancestry born during 1955–1993 in Guatemala (*n*_observations_ 157 067)

Summary statistics of the SITAR models show that the fixed effects (size, timing and intensity) explained between 69·0 and 98·3 % of variance in the outcomes, with the model for height being the most accurate (Table 3). Figure 1 summarises the mean height-for-age curve for the full sample and each sex. Correlations between the size, timing and intensity fixed effects were small to modest (0·17–0·44), except for grip strength where the size and intensity correlation coefficient was higher at 0·81 (Table 3).

**Table 3:** Median age at peak growth velocity and Super-Imposition by Translation and Rotation (SITAR) model summaries, including number of individuals (*n*) and parameter sd for height, weight, BMI, grip strength fitted on age by gender, in the sample of children and adolescents (*n* 40 484) aged 3–19 years of Ladino and Maya ancestry born during 1955–1993 in Guatemala (*n*_observations_ 157 067)

Variable	SITAR model summary	Males	Females
Height (cm)	*n*	22 746	17 738
	Median age at peak velocity (years)	13·3	11·4
	Spline degrees of freedom	6	6
	sd of size random effect (cm)	8·0	7·1
	sd of timing random effect (%)	7·9	8·5
	sd of intensity random effect (%)	12·1	12·4
	Timing–intensity correlation	0·37	0·17
	Size–timing correlation	0·33	0·22
	Size–intensity correlation	0·64	0·48
	Variance explained (%)	97·8	98·2
Weight (kg)	*n*	22 734	17 720
	Median age at peak velocity (years)	13·2	11·8
	Spline degrees of freedom	5	5
	sd of size random effect (%)	15·1	14·2
	sd of timing random effect (years)	1·0	1·0
	sd of intensity random effect (%)	15·2	16·4
	Timing-intensity correlation	−0·45	−0·21
	Size–timing correlation	−0·07	−0·02
	Size–intensity correlation	0·33	0·23
	Variance explained (%)	93·7	94·3
BMI (kg/m^2^)	*n*	22 734	17 720
	Median age at peak velocity (years)	14·6	12·2
	Spline degrees of freedom	4	4
	sd of size random effect (%)	12·0	12·0
	sd of intensity random effect (%)	33·2	36·2
	Size–intensity correlation	0·45	0·33
	Variance explained (%)	84·7	84·9
Grip strength (kg-force)	*n*	22 716	17 695
	Median age at peak velocity (years)	14·4	11·8
	Spline degrees of freedom	5	4
	sd of size random effect (kg-force)	3·4	3·1
	sd of intensity random effect (%)	23·9	24·8
	Size–intensity correlation	0·81	0·81
	Variance explained (%)	77·7	69·0

### Height

#### Size

**Fig. 2:**
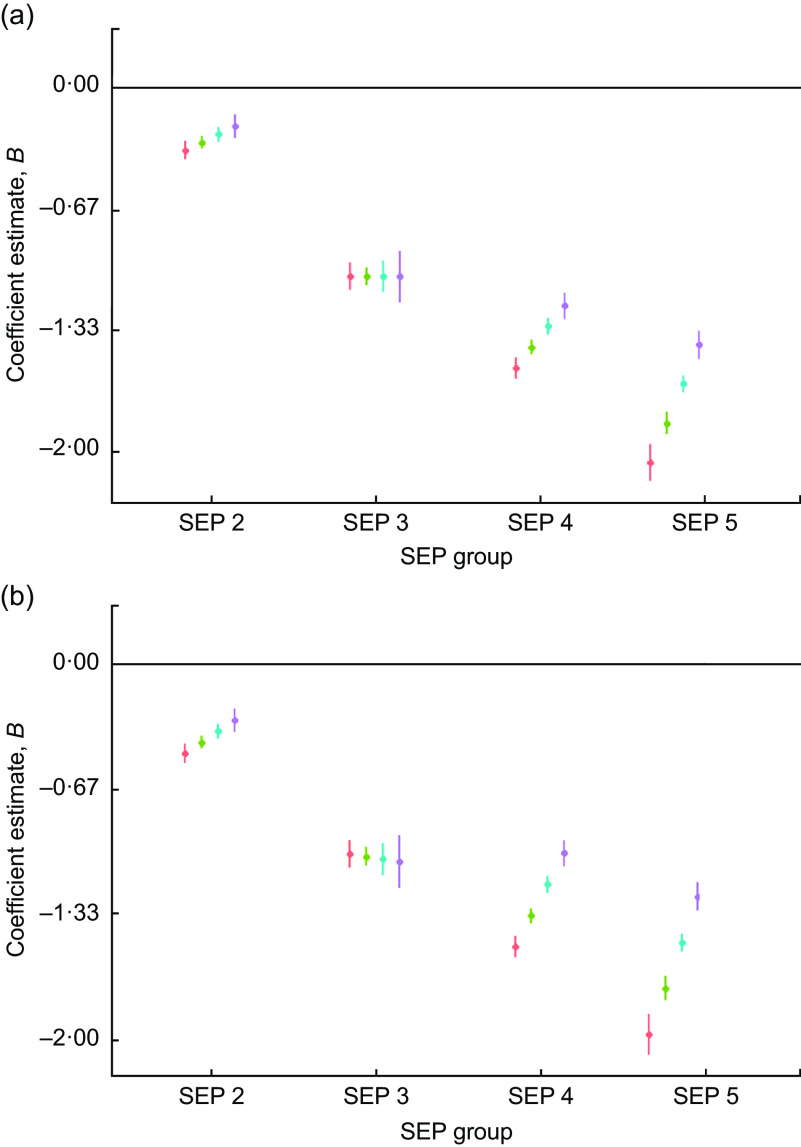
Regression estimates of (a) male and (b) female height size *Z*-scores (dots) with 95 % CI (whiskers) across socio-economic position (SEP) groups (2–5), with SEP 1 as reference group (horizontal line at 0), by decade of birth centred on 1960 (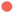), 1970 (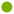), 1980 (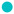) and 1990 (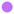), for children and adolescents (*n* 40 484) aged 3–19 years of Ladino and Maya ancestry born during 1955–1993 in Guatemala (*n*_observations_ 157 067). The plots were created using R packages ggplot2, broom, jtools, dotwhisker and dplyr (see the online supplementary material for references)

There was evidence of high inequality in height *Z*-scores in this population whereby the lowest-SEP males were, on average, −1·8 (95 % CI −1·8, −1·7) sd and the females were −1·6 (95 % CI −1·7, −1·6) sd shorter relative to the reference group (SEP 1) at the centre of the date of birth distribution (in year 1972). Figure 2 shows the regression coefficients of male and female height *Z*-scores by SEP group and through the date of birth range. There is a narrowing of inequality through time, whereby by 1990 the two lowest-SEP groups have very similar height *Z*-scores to SEP 3. However, these three groups remain over 1 sd below SEP 1 in both male and female height (Fig. 2). The interaction estimates show formal evidence that the SEP inequality has decreased for both males and females as the SEP groups 4 and 5 show larger secular increases in height than the other groups (Table 4).

**Table 4:** Socio-economic position (SEP) group and decimal date of birth (DOB, in decades) regressions (regression coefficient *B*, se and *P* value) for males and females in height, weight, BMI and grip strength size *Z*-scores, with SEP 1 as the reference group, in the sample of children and adolescents (*n* 40 484) aged 3–19 years of Ladino and Maya ancestry born during 1955–1993 in Guatemala (*n*_observations_ 157 067)

Dependent variable	Predictor	Males			Females		
		*B*	se	*P* value	*B*	se	*P* value
Height size *Z*-score	SEP 2	−0·3	0·02	<0·0001	−0·4	0·01	<0·0001
	SEP 3	−1·0	0·03	<0·0001	−1·0	0·02	<0·0001
	SEP 4	−1·4	0·02	<0·0001	−1·3	0·02	<0·0001
	SEP 5	−1·8	0·03	<0·0001	−1·7	0·03	<0·0001
	DOB × SEP 1	−0·02	0·02	0·06	−0·07	0·01	<0·0001
	DOB × SEP 2	0·04	0·02	0·005	0·06	0·02	0·0003
	DOB × SEP 3	−0·00	0·03	0·98	−0·01	0·03	0·7
	DOB × SEP 4	0·1	0·02	<0·0001	0·2	0·02	<0·0001
	DOB × SEP 5	0·2	0·03	<0·0001	0·2	0·03	<0·0001
		*R* ^2^ adjusted = 0·34			*R* ^2^ adjusted = 0·34		
Weight size *Z*-score	SEP 2	−0·1	0·02	<0·0001	−0·07	0·02	0·0003
	SEP 3	−0·8	0·03	<0·0001	−0·6	0·03	<0·0001
	SEP 4	−1·1	0·02	<0·0001	−0·8	0·02	<0·0001
	SEP 5	−1·4	0·03	<0·0001	−1·0	0·03	<0·0001
	DOB × SEP 1	0·003	0·001	0·02	−0·03	0·02	0·03
	DOB × SEP 2	0·1	0·02	<0·0001	0·1	0·02	<0·0001
	DOB × SEP 3	−0·03	0·03	0·4	−0·02	0·04	0·6
	DOB × SEP 4	0·1	0·02	<0·0001	0·2	0·02	<0·0001
	DOB × SEP 5	0·2	0·03	<0·0001	0·2	0·03	<0·0001
		*R* ^2^ adjusted = 0·23			*R* ^2^ adjusted = 0·16		
BMI size *Z*-score	SEP 2	0·1	0·02	<0·0001	0·2	0·02	<0·0001
	SEP 3	−0·3	0·03	<0·0001	−0·1	0·04	0·005
	SEP 4	−0·4	0·02	<0·0001	−0·2	0·02	<0·0001
	SEP 5	−0·5	0·03	<0·0001	−0·2	0·04	<0·0001
	DOB × SEP 1	0·008	0·002	<0·0001	0·03	0·02	0·052
	DOB × SEP 2	0·1	0·02	0·0001	0·1	0·02	<0·0001
	DOB × SEP 3	−0·1	0·04	0·006	−0·06	0·04	0·1
	DOB × SEP 4	0·04	0·02	0·077	0·1	0·02	<0·0001
	DOB × SEP 5	0·1	0·03	0·009	0·1	0·03	0·011
		*R* ^2^ adjusted = 0·07			*R* ^2^ adjusted = 0·04		
Grip strength size *Z*-score	SEP 2	−0·2	0·02	<0·0001	−0·3	0·02	<0·0001
	SEP 3	−0·9	0·03	<0·0001	−0·7	0·03	<0·0001
	SEP 4	−1·1	0·02	<0·0001	−0·9	0·02	<0·0001
	SEP 5	−1·4	0·03	<0·0001	−1·3	0·03	<0·0001
	DOB × SEP 1	−0·003	0·001	0·03	−0·04	0·01	0·016
	DOB × SEP 2	0·00	0·02	0·8	0·03	0·02	0·1
	DOB × SEP 3	−0·01	0·04	0·8	−0·1	0·04	0·025
	DOB × SEP 4	0·1	0·02	<0·0001	0·1	0·02	<0·0001
	DOB × SEP 5	0·3	0·03	<0·0001	0·3	0·03	<0·0001
		*R* ^2^ adjusted = 0·22			*R* ^2^ adjusted = 0·16		

### Weight

#### Size

**Fig. 3:**
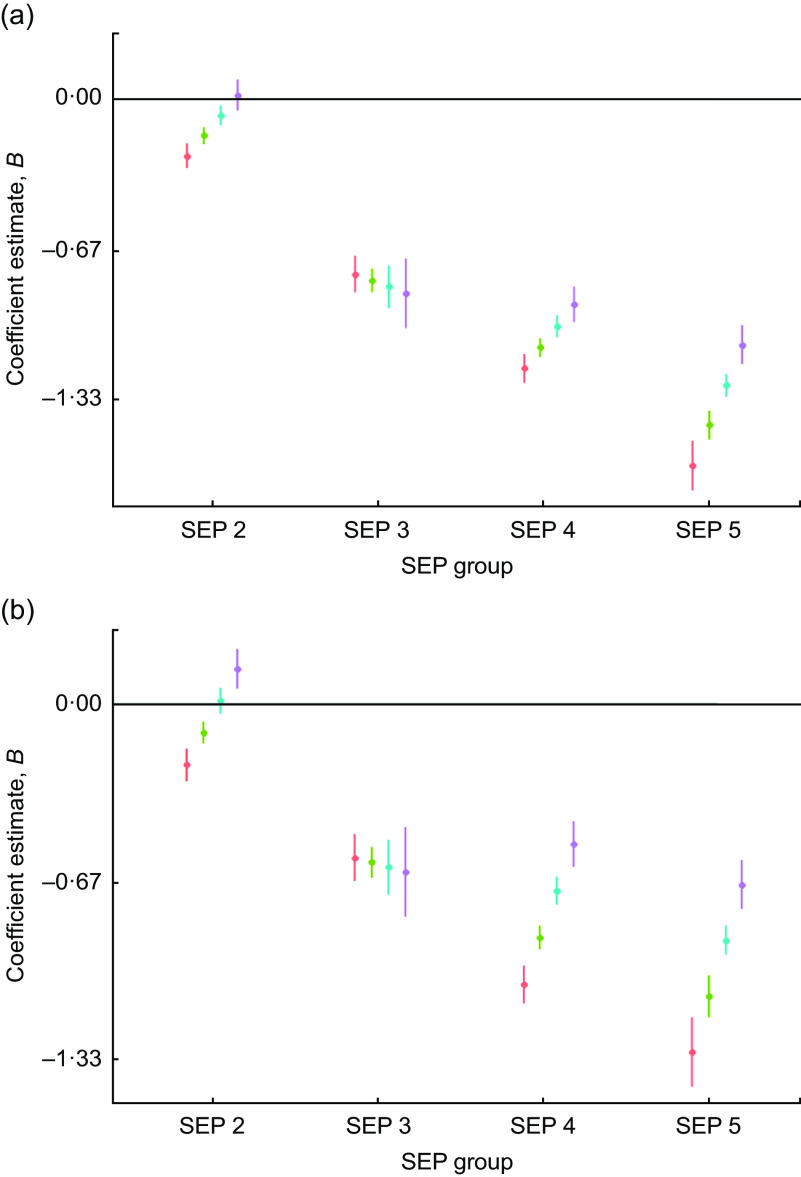
Regression estimates of (a) male and (b) female weight size *Z*-scores (dots) with 95 % CI (whiskers) across socio-economic position (SEP) groups (2–5), with SEP 1 as reference group (horizontal line at 0), by decade of birth centred on 1960 (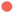), 1970 (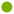), 1980 (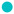) and 1990 (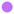), for children and adolescents (*n* 40 484) aged 3–19 years of Ladino and Maya ancestry born during 1955–1993 in Guatemala (*n*_observations_ 157 067). The plots were created using R packages ggplot2, broom, jtools, dotwhisker and dplyr (see the online supplementary material for references)

The inequality between the highest and lowest SEP in weight at the centre of the date of birth distribution was −1·4 (95 % CI −1·4, −1·3) sd for males and −1·0 (95 % CI −1·1, −0·9) sd for females. Figure 3 indicates that there was a narrowing of the inequality through time, and the regression estimates of the SEP and date of birth interactions confirm this (Table 4). As with height, the narrowing inequality is due to faster secular increases in weight for SEP groups 4 and 5.

### BMI

#### Size

**Fig. 4:**
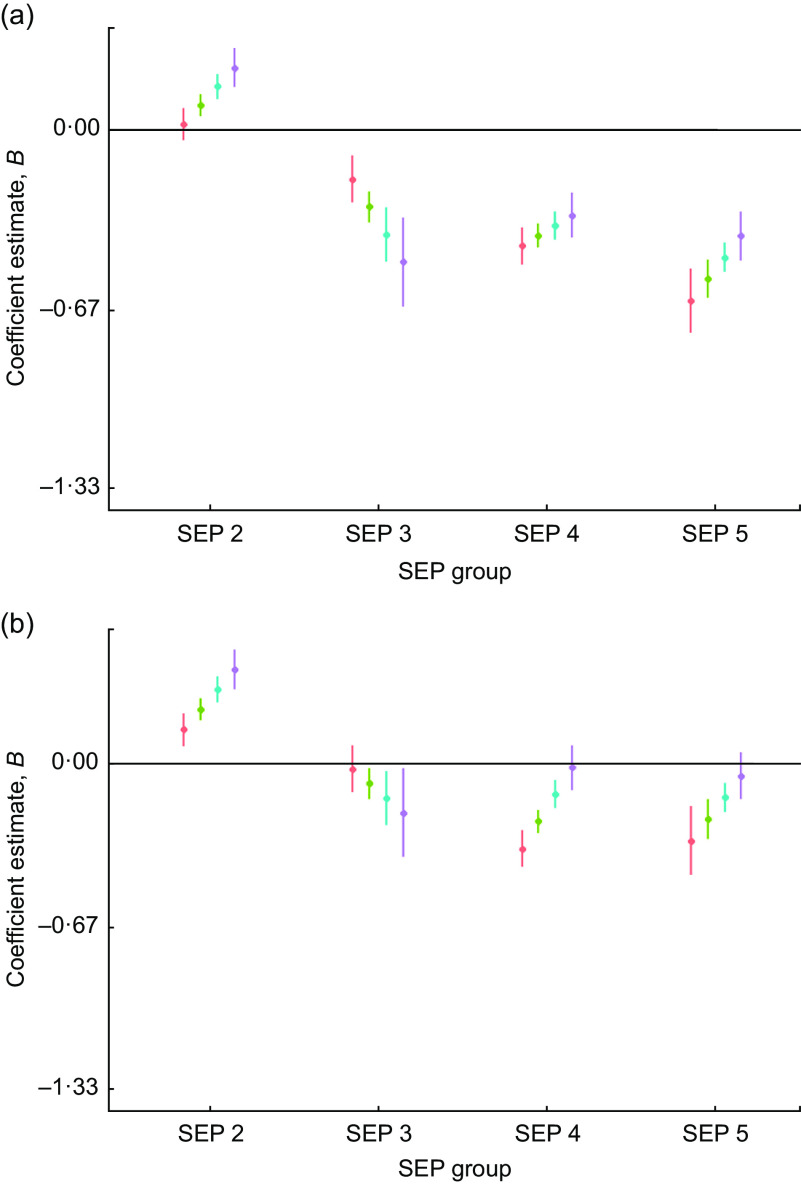
Regression estimates of (a) male and (b) female BMI size *Z*-scores (dots) with 95 % CI (whiskers) across socio-economic position (SEP) groups (2–5), with SEP 1 as reference group (horizontal line at 0), by decade of birth centred on 1960 (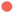), 1970 (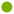), 1980 (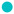) and 1990 (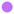), for children and adolescents (*n* 40 484) aged 3–19 years of Ladino and Maya ancestry born during 1955–1993 in Guatemala (*n*_observations_ 157 067). The plots were created using R packages ggplot2, broom, jtools, dotwhisker and dplyr (see the online supplementary material for references)

Inequalities in BMI were, on average, small compared with height and weight, the difference between the highest and lowest SEP was −0·5 (95 % CI −0·6, −0·5) sd for males and −0·2 (95 % CI −0·3, −0·1) sd for females. There were significant secular increases in BMI for males of SEP groups 1, 2, 4 and 5, and females of SEP groups 2, 4 and 5 (Table 4, Fig. 4). In SEP 3 males, the BMI trend was negative (Table 4).

### Grip strength

#### Size

**Fig. 5:**
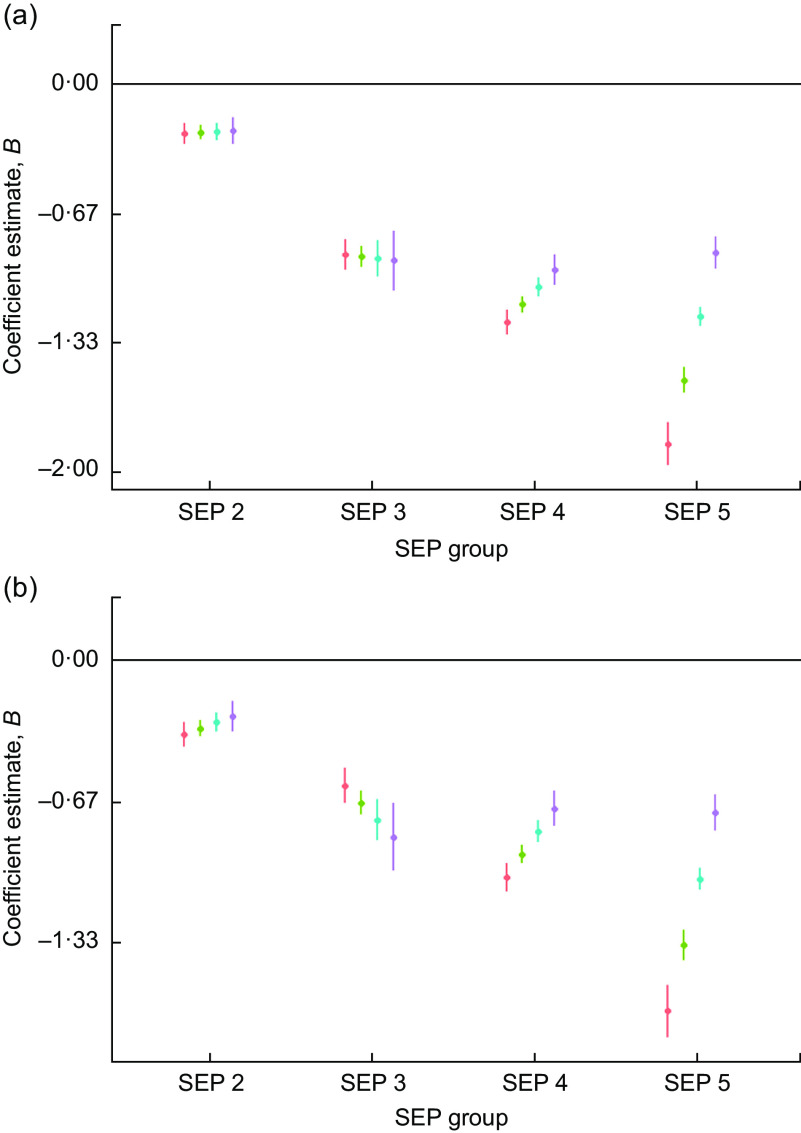
Regression estimates of (a) male and (b) female grip strength size *Z*-scores (dots) with 95 % CI (whiskers) across socio-economic position (SEP) groups (2–5), with SEP 1 as reference group (horizontal line at 0), by decade of birth centred on 1960 (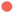), 1970 (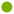), 1980 (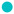) and 1990 (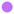), for children and adolescents (*n* 40 484) aged 3–19 years of Ladino and Maya ancestry born during 1955–1993 in Guatemala (*n*_observations_ 157 067). The plots were created using R packages ggplot2, broom, jtools, dotwhisker and dplyr (see the online supplementary material for references)

There was evidence of high inequality in grip strength *Z*-scores whereby the lowest-SEP males were, on average, −1·44 (95 % CI −1·50, −1·38) sd and the females were −1·3 (95 % CI −1·3, −1·2) sd weaker than the reference group (SEP 1). There was a narrowing of inequality in grip strength through time, particularly for SEP 5 individuals whose grip strength increased, on average, by 0·3 (95 % CI 0·3, 0·4) sd per decade of birth for males and by 0·3 (95 % CI 0·3, 0·4) sd per decade of birth for females (Table 4, Fig. 5). By 1990, SEP groups 3, 4 and 5 had very similar grip strength, albeit still close to 1 sd lesser than the grip of SEP 1 (Fig. 5).

### Timing and intensity (age at peak growth velocity and peak growth velocity)

Males in SEP 5 had a 0·2 (95 % CI 0·2, 0·3) sd and females a 0·4 (95 % CI 0·4, 0·5) sd later age at peak height velocity than individuals of SEP 1, while individuals in SEP 2 had earlier height APV than SEP 1 (see online supplementary material, Supplemental Table S1 and Supplemental Fig. S1). APV for both males and females of SEP groups 3–5 seemed to converge, whereby APV increased for SEP 3 and decreased for SEP 4 and 5 through time (Supplemental Table S1, Supplemental Fig. S1). PV showed larger SEP differences than APV, each decrease in SEP group resulted in a decrease in PV of 0·2–0·4 sd in males and 0·2–0·4 sd in females (Supplemental Table S2, Supplemental Figs S2 and S3). Secular increases in height PV were observed for SEP groups 2–5 (Supplemental Table S2, Supplemental Figs S2 and S3).

In weight, there were larger inequalities in APV than for height. The APV for SEP 5 was 0·9 (95 % CI 0·8, 0·9) sd delayed compared with SEP 1 males and 0·9 (95 % CI 0·8, 1·0) sd delayed for SEP 5 females compared with SEP 1 females (see online supplementary material, Supplemental Table S1 and Supplemental Fig. S1). As with height, individuals in SEP 2 had earlier weight APV than individuals from SEP 1 (Supplemental Table S1). Weight PV showed smaller inequalities than height PV (Supplemental Table S2, Supplemental Figs S2 and S3). Secular increases in peak weight velocity were observed for SEP groups 1, 2, 4 and 5 (Supplemental Table S2, Supplemental Figs S2 and S3).

The PV of BMI gain was highest in both males and females of SEP 2, and for females, all SEP groups had a higher PV in BMI than the reference group (see online supplementary material, Supplemental Table S2 and Supplemental Figs S2 and S3). There were significant secular increases in BMI PV for females of SEP 2 and 4, and for males of SEP 2, 4 and 5 (Supplemental Table S2, Supplemental Figs S2 and S3).

Grip intensity showed very similar patterns to grip size, whereby the initially large inequalities in grip PV decreased through time for both males and females (see online supplementary material, Supplemental Table S2 and Supplemental Figs S2 and S3).

## Discussion

Using data on over 40 000 children, the present study found that considerable differences in height, weight and grip strength remain between the richest and the poorest in Guatemala, despite general decreases in inequality.

Overall, our findings are supportive of previous work on within-population SEP differences in growth that found patterns of secular changes differ across SEP group^(8)^. However, the scale of the reported SEP differences in the present sample is considerably larger, and to our knowledge, we are the first to report SEP differences for timing and intensity of growth for weight, BMI and grip strength. In height the lowest-SEP individuals were 2 sd below the highest in year 1960, and the comparison with the WHO mean *Z*-scores by SEP group confirmed that, overall, this group was stunted by conventional height-for-age *Z*-score cut-offs. As previously stated, the high-SEP reference group of our study differs by only 0·2 *Z*-scores from international references in mean height and can be thus considered healthy or ‘normal’ height. Still, recent reports show that even among the highest 20 % of the Guatemalan income distribution, stunting rates of 17 % have been reported, highlighting the pernicious effects of the environment for growth in Guatemala across SEP groups^(12)^.

The lowest two SEP groups showed an overall increase in body size, likely reflecting a raised standard of living and more favourable early-life environments. The secular changes coincided with the internal conflict in Guatemala which started in the 1950s, became a civil war in 1960 and continued, with varying intensity, until the signing of the peace accords in 1996. While the effects of the conflict were devastating to a large part of the civilian population in the rural countryside, particularly the Maya, reports suggest Guatemala City was better buffered^(10)^. The two Maya schools were located in a village 25 km from Guatemala City and this proximity may have buffered the village as well. Between 1960 and 1985, American and European international corporations established offices in the City and the period included steady economic growth^(22)^. This overall economic growth may have supported the physical growth of the lower SEP, despite the ongoing conflict. From 1974 to 1990 the country experienced the most severe consequences of the civil war resulting in an economic recession and the government becoming bankrupt by 1990. Previous work with smaller samples sizes found that 10–11-year-olds of SEP groups 1, 2 and 3 born between 1974 and 1985 experienced a negative effect on height^(23)^. We did not find evidence for this, except for SEP 1 females who showed an overall negative secular trend in height. Why this trend existed only for this group is unknown and overall our results suggest secular increases in height.

The Cold War US investment in Guatemala included fast-food and supermarket chains. The urban population’s access to ‘Western’ nutrition was increased, although the prices of many imported food items remain high even today. The Happy Meal, McDonald’s famous children’s meal, was invented in Guatemala in the mid-1970s before being launched worldwide^(24)^. The secular trends we observed in the anthropometric outcomes of the SEP 2 individuals (upper-middle economic class) fit well with these changes to the food environment, as this group is likely to have been able to afford the newly available, energy-rich diets. The SEP group 1 (highest economic class) showed a very modest secular increase in male weight and a small negative trend in female weight, perhaps suggesting that the change in the nutritional landscape and access to energy-dense foods did not change their lifestyle significantly. For BMI, SEP 1 had the smallest secular increases of all the groups. There is evidence in the literature of high-SEP individuals from the USA and France having lower rates of obesity compared with lower-SEP groups^(25)^, possibly relating to social desirability of non-overweight body shape and better awareness among high-SEP individuals of healthy behaviours due to higher educational attainment. In the SEP groups 4 and 5 there were secular increases in BMI for both males and females; however, they remained, on average, with lower BMI than that of their counterparts in SEP 1 and 2 at the end of the studied period. In SEP 3 there was a secular decrease in male BMI and no change for females. The reason for this is unclear but due to the ‘middle SEP’ nature of the two schools included in this category, it is possible that the socio-economic composition of the students in this group changed through time. If so, this could explain why there was very little evidence of secular trend in any parameter for this SEP group.

To try to investigate physical function in the sample, we looked at the longitudinal grip strength measure. No previous work, to our knowledge, has modelled childhood grip strength data in such a large sample, and there are no grip strength references based on longitudinal data for under 18-year-olds. The inequality in grip strength was similar to that in height, implying that height differences between SEP groups are reflected in the characteristics measured by grip strength: lean mass, especially muscle mass, and overall body strength. The analysis found that taller children are, on average, stronger. An international comparison of grip strength and BMI data concluded that both overweight and obesity, as well as chronic undernourishment, are associated with lower grip strength^(26)^. In the present study, individuals from SEP groups 4 and 5 increased their grip strength through time, which supports a positive association between height, weight and grip strength, and by implication lean mass. Indigenous school youths aged 6–17 years in Oaxaca, southern Mexico, were surveyed in 1968, 1978 and 2000, and showed small and generally non-significant increases in grip strength. The small secular gains in muscular strength were generally proportional to secular gains in body weight and height^(27)^. For the UVG Study, SEP groups 1, 2 and 3 showed no secular increase in grip strength, despite increases in height and weight for SEP 2. The lack of association might reflect a change towards a more sedentary lifestyle, a phenomenon which has been globally well recorded in recent decades^(28)^. Similar lack of secular increase in adolescent grip strength has been found in Canada and the USA, despite increasing adolescent body weights^(29)^. In adults, there has been a reported secular decrease in grip strength^(30)^. In light of these other studies, we interpret the findings of our analysis for grip strength to indicate that for SEP groups 4 and 5, the increase in body weight and BMI with time was due more to the accrual of lean mass (muscle, bone, organs) than to fat mass.

At the end of the studied period, the lowest three SEP groups remained approximately 1 sd below the highest in height, weight and grip strength, signalling that even in the presence of secular increases, large inequalities persist. It is not feasible to pinpoint the exact reason for this, but these findings are likely related to the continuing disparities in Guatemala in access to health care, education and employment^(31)^. The lower-SEP groups also face insecurities in virtually all aspects of daily life (food, water, electricity, government services, high crime) due to poverty. Guatemala has the highest rate of poverty in all Latin America^(32)^. The public health-care and educational systems are severely underfunded, and the country has experienced a continuous procession of corruption scandals including government officials involved in organised crime networks. Until these underlying structural inequalities are addressed, it is possible that the health and growth discrepancies will remain high.

Investigation of timing (APV) and intensity (PV) of growth revealed further inequalities, whereby the richer individuals had both earlier maturity in height and weight, and a higher peak growth velocity. Peak weight gain followed peak height gain in females but not males. It is unclear which environmental conditions could result in shifts in growth timing and intensity, and whether growth faltering could be prevented by interventions targeted at the ‘speed’ or timing of growth. Evidence does suggest both timing and intensity of growth are to some extent influenced by intra-uterine growth, hormone balance (particularly insulin-like growth factor-I), nutritional status including calcium intake, and obesity status^(32–36)^. Early puberty is associated with increased risk of later-life cardiovascular mortality, hypertension, metabolic syndrome and mental health problems^(37–39)^. Late puberty is commonly thought to reflect poor early-life growth environment and is associated with poorer bone health in early old age^(37,40,41)^. This raises the question whether there is an ‘ideal’ APV and PV in terms of risk for later-life health and disease outcomes. The presence of socio-economic inequalities and secular shifts in these variables suggests a potentially significant environmental effect. In height, inequalities were primarily reflected in lower peak velocity for low-SEP individuals and less so in differences in pubertal timing (i.e. the more disadvantaged children grew more slowly), whereas for weight, the inequalities manifested more in an older age at peak weight gain for SEP groups 4 and 5. In female BMI, all SEP groups had a higher intensity of growth throughout the studied period than the reference group. As discussed above, this may reflect social discrepancies in weight desirability and body image *v*. the socio-economic and health-based height inequalities. Trends towards increasing weight-for-height in SEP group 2 in particular could in the future result in negative health outcomes from metabolic syndrome and its related diseases, including diabetes and CVD, for those individuals whose BMI is in the overweight or obese range^(42)^.

Our study had several strengths, including the UVG’s data set of Guatemalan anthropometry and the SITAR method of analysis that incorporates information on size, timing and intensity of growth. The data, collected over four decades, span nearly the entire SEP range of Guatemala and represent individuals of both Ladino and Maya ethnicity. A limitation of the present study is that as the data collection extended only until 1999, we are unable to describe more recent changes in growth or other outcomes. Further, our study cannot disentangle a possible ethnicity-linked influence in growth measures for SEP 5 which comprises predominantly Maya children, who are known to be shorter than Ladinos^(12)^. Differences of growth measures between SEP 5 and SEP 1–4 are due to a wide range of factors associated with school attended and ethnicity. We cannot disentangle the two because there are very few Maya in SEP 1–4, or Ladinos in SEP 5. Over the last 500 years, the Maya have been both socially and politically repressed, and continue to have a higher incidence of poverty compared with the Ladino population^(31,43–45)^. We doubt that genetics plays a role in the short stature of the Maya because we know that Maya 5–12-year-olds growing up in the USA are, on average, 11 cm taller than the Maya of the same ages living in Guatemala^(19)^. Finally, we did not include estimates of body fat in the analyses. A child with high BMI-for-age may be ‘fat’, muscular, or an early maturer compared with the population mean used to create the reference. We did, however, infer changes in fatness and lean mass via the data for height, weight and grip strength. Our data encompassed only urban and semi-urban Guatemalans, which means that inequalities in the growth patterns of the rural population could not be assessed. The semi-urban Maya sample may include segments of the more rural population who migrated to live closer to Guatemala City over the course of the civil war.

## Conclusions

There has been a narrowing of disparity in physical growth between socio-economic status groups in Guatemala, but stark inequality remains. This is likely to result in poorer population health and productivity, as particularly low adult height has been connected to lower human capital. The later-life health implications of the increasingly fast pubertal BMI gains across the SEP groups remain to be investigated. While current follow-up studies in Guatemala focus only on rural, low-SEP Ladinos, most individuals in our samples of Maya and higher-SES Ladinos can be expected to be alive and currently aged 25–76 years old. This could facilitate future longitudinal follow-ups, for example looking at life-course disease risk factors, on urban and Maya samples, ranging from very high to very low SEP, whose childhood growth patterns are now well defined.

## Supplementary material

For supplementary material accompanying this paper visit https://doi.org/10.1017/S1368980019003239. click here to view supplementary material

## References

[1] Kuh D , Richards M , Cooper R et al. (2014) Life course epidemiology, ageing research, and maturing cohort studies: a dynamic combination for understanding healthy ageing. In A Life Course Approach to Healthy Ageing, pp. 3–15 [ D Kuh , R Cooper , R Hardy et al., editors]. Oxford: Oxford University Press.

[2] NCD Risk Factor Collaboration (2016) A century of trends in adult human height. Elife 5, e13410. 27458798 10.7554/eLife.13410 PMC4961475

[3] Victora CG , Adair L , Fall C et al. (2008) Maternal and child undernutrition: consequences for adult health and human capital. Lancet 371, 340–357. 18206223 10.1016/S0140-6736(07)61692-4 PMC2258311

[4] United Nations (2018) Sustainable Development Knowledge Platform. Sustainable Development Goals. https://sustainabledevelopment.un.org/sdgs (accessed October 2018).

[5] Bogin B (1999) Patterns of Human Growth. Cambridge: Cambridge University Press.

[6] Vollmer S , Harttgen K , Subramanyam MA et al. (2014) Association between economic growth and early childhood undernutrition: evidence from 121 Demographic and Health Surveys from 36 low-income and middle-income countries. Lancet Glob Health 2, e225–e234. 25103063 10.1016/S2214-109X(14)70025-7

[7] Tanner J (1989) Foetus into Man: Physical Growth from Conception to Maturity. Brickendonbury: Castlemead Publications.

[8] Bann D , Johnson W , Li L et al. (2018) Socioeconomic inequalities in childhood and adolescent body-mass index, weight, and height from 1953 to 2015: an analysis of four longitudinal, observational, British birth cohort studies. Lancet Public Health 3, e194–e203. 29571937 10.1016/S2468-2667(18)30045-8 PMC5887082

[9] Marini A & Gragnolati M (2003) Malnutrition and Poverty in Guatemala. Washington, DC: World Bank.

[10] Tomuschat C , Lux De Coti O & Balsells Tojo A (1999) Guatemala: Memory of Silence. Report of the Commission for Historical Clarification Conclusions and Recommendations. https://hrdag.org/wp-content/uploads/2013/01/CEHreport-english.pdf (accessed November 2018).

[11] Martinez B , Webb MF , Gonzalez A et al. (2018) Complementary feeding intervention on stunted Guatemalan children: a randomised controlled trial. BMJ Paediatr 2, e000213. 10.1136/bmjpo-2017-000213 PMC5926563 29719876

[12] Ministerio de Salud Pública y Asistencia Social, Instituto Nacional de Estadística, Secretaría de Planificación y Programación de la Presidencia et al . (2017) Encuesta Nacional de Salud Materno Infantil 2014–2015: Informe Final. Guatemala City: MSPAS/INE/Segeplán/ICF International.

[13] Martorell R , Habicht J-P & Rivera JA (1995) History and design of the INCAP longitudinal study (1969–77) and its follow-up (1988–89). J Nutr 125, 1027–1041. 10.1093/jn/125.suppl_4.1027S 7536830

[14] Bogin B , Camacho de Paz R & MacVean RB (2018) The Universidad del Valle de Guatemala Longitudinal Study of Child and Adolescent Development: History, Description, and Research Prospectus. Guatemala City: Universidad del Valle de Guatemala.

[15] Bogin B & MacVean RB (1983) The relationship of socioeconomic status and sex to body size, skeletal maturation, and cognitive status of Guatemala City schoolchildren. Child Dev 54, 115–128. 6831980

[16] Hoddinott J , Maluccio JA , Behrman JR et al. (2008) Effect of a nutrition intervention during early childhood on economic productivity in Guatemalan adults. Lancet 371, 411–416. 18242415 10.1016/S0140-6736(08)60205-6

[17] Maluccio J , Hoddinott J , Behrman J et al. (2006) The Impact of Nutrition During Early Childhood on Education among Guatemalan Adults. PSC Working Paper Series no. 06-04. Philadelphia, PA: Population Studies Center, University of Pennsylvania.

[18] Bogin B & MacVean RB (1978) Growth in height and weight of urban Guatemalan primary school children of low and high socioeconomic class. Hum Biol 50, 477–487. 744589

[19] Bogin B , Smith P , Orden AB et al. (2002) Rapid change in height and body proportions of Maya American children. Am J Hum Biol 14, 753–761. 12400036 10.1002/ajhb.10092

[20] Cole TJ , Donaldson MDC & Ben-Shlomo Y (2010) SITAR – a useful instrument for growth curve analysis. Int J Epidemiol 39, 1558–1566. 20647267 10.1093/ije/dyq115 PMC2992626

[21] Simpkin AJ , Sayers A , Gilthorpe MS et al. (2017) Modelling height in adolescence: a comparison of methods for estimating the age at peak height velocity. Ann Hum Biol 44, 715–722. 29113497 10.1080/03014460.2017.1391877 PMC5743008

[22] R Core Team (2018) R: A Language and Environment for Statistical Computing. Vienna: R Foundation for Statistical Computing; available at http://www.r-project.org/ (accessed November 2018).

[23] Trading Economics (2018) Guatemala GDP 1960–2018. https://tradingeconomics.com/guatemala/gdp (accessed October 2018).

[24] Bogin B & Keep R (1999) Eight thousand years of economic and political history in Latin America revealed by anthropometry. Ann Hum Biol 26, 333–351. 10462154 10.1080/030144699282651

[25] El Periodico (2006) La señora del Mac menú. https://web.archive.org/web/20140203041617/http://www.elperiodico.com.gt/es/20060801/14/30397/ (accessed November 2018).

[26] Drewnowski A , Moudon AV , Jiao J et al. (2014) Food environment and socioeconomic status influence obesity rates in Seattle and in Paris. Int J Obes (Lond) 38, 306–314. 23736365 10.1038/ijo.2013.97 PMC3955164

[27] Massy-Westropp NM , Gill TK , Taylor AW et al. (2011) Hand grip strength: age and gender stratified normative data in a population-based study. BMC Res Notes 4, 127. 21492469 10.1186/1756-0500-4-127 PMC3101655

[28] Malina RM , Reyes MEP , Tan SK et al. (2010) Secular change in muscular strength of indigenous rural youth 6–17 years in Oaxaca, southern Mexico: 1968–2000. Ann Hum Biol 37, 168–184. 20141482 10.3109/03014460903325193

[29] Popkin BM , Adair LS & Ng SW (2012) Global nutrition transition and the pandemic of obesity in developing countries. Nutr Rev 70, 3–21. 22221213 10.1111/j.1753-4887.2011.00456.x PMC3257829

[30] Silverman IW (2011) The secular trend for grip strength in Canada and the United States. J Sports Sci 29, 599–606. 21391084 10.1080/02640414.2010.547209

[31] Silverman IW (2015) Age as a moderator of the secular trend for grip strength in Canada and the United States. Ann Hum Biol 42, 199–209. 25041340 10.3109/03014460.2014.934920

[32] Cabrera M , Lustig N & Morán HE (2015) Fiscal policy, inequality, and the ethnic divide in Guatemala. World Dev 76, 263–279.

[33] Cole TJ , Ahmed ML , Preece MA et al. (2015) The relationship between Insulin-like growth factor 1, sex steroids and timing of the pubertal growth spurt. Clin Endocrinol (Oxf) 82, 862–869. 25418044 10.1111/cen.12682 PMC4949545

[34] De Leonibus C , Marcovecchio ML , Chiavaroli V et al. (2014) Timing of puberty and physical growth in obese children: a longitudinal study in boys and girls. Pediatr Obes 9, 292–299. 23713062 10.1111/j.2047-6310.2013.00176.x

[35] Karaolis-Danckert N , Buyken AE , Sonntag A et al. (2009) Birth and early life influences on the timing of puberty onset: results from the DONALD (DOrtmund Nutritional and Anthropometric Longitudinally Designed) study. Am J Clin Nutr 90, 1559–1565. 19828713 10.3945/ajcn.2009.28259

[36] Ward KA , Cole TJ , Laskey MA et al. (2014) The effect of prepubertal calcium carbonate supplementation on skeletal development in Gambian boys – a 12-year follow-up study. J Clin Endocrinol Metab 99, 3169–3176. 24762110 10.1210/jc.2014-1150 PMC5165037

[37] Wehkalampi K , Silventoinen K , Kaprio J et al. (2008) Genetic and environmental influences on pubertal timing assessed by height growth. Am J Hum Biol 20, 417–423. 18293372 10.1002/ajhb.20748 PMC3769165

[38] Patton GC & Viner R (2007) Pubertal transitions in health. Lancet 369, 1130–1139. 17398312 10.1016/S0140-6736(07)60366-3

[39] Prentice P & Viner RM (2013) Pubertal timing and adult obesity and cardiometabolic risk in women and men: a systematic review and meta-analysis. Int J Obes (Lond) 37, 1036–1043. 23164700 10.1038/ijo.2012.177

[40] Viner R , Ross D , Hardy R et al. (2015) Life course epidemiology: recognising the importance of adolescence. J Epidemiol Community Health 69, 719–720. 25646208 10.1136/jech-2014-205300 PMC4515995

[41] Cole TJ & Mori H (2018) Fifty years of child height and weight in Japan and South Korea: contrasting secular trend patterns analyzed by SITAR. Am J Hum Biol 30, e23054. 28833849 10.1002/ajhb.23054 PMC5811819

[42] Kuh D , Muthuri SG , Moore A et al. (2016) Pubertal timing and bone phenotype in early old age: findings from a British birth cohort study. Int J Epidemiol 45, 1113–1124. 27401728 10.1093/ije/dyw131 PMC5075580

[43] World Health Organization (2018) Obesity and overweight. https://www.who.int/news-room/fact-sheets/detail/obesity-and-overweight (accessed September 2018).

[44] Lovell WG & Lutz CH (1996) ‘A dark obverse’: Maya survival in Guatemala: 1520–1994. Am Geogr Soc 86, 398–407.

[45] Lovell WG (2010) A Beauty That Hurts: Life and Death in Guatemala, pp. 105–120. Austin, TX: University of Texas Press.

[46] de Onis M , Onyango A , Borghi E et al. (2007) Development of a WHO growth reference for school-aged children and adolescents. Bull World Health Organ 85, 661–666. 10.2471/BLT.07.043497 PMC2636412 18026621

