# Supplementary material for: Four decades of socio-economic inequality and secular change in the physical growth of Guatemalans
Source: Public Health Nutr. 2019 Dec 5;23(8):1381–91. doi: 10.1017/S1368980019003239 (PMC7196735; doi:10.1017/S1368980019003239)
Supplement: Supplementary file 1 [file S1368980019003239sup001.docx]

Supplementary material

| **Males** | | | | **Females** | | | |
| --- | --- | --- | --- | --- | --- | --- | --- |
| **Dependent Variable** | **Predictor** | **B** | **Std. Error** | **P-value** | **B** | **Std. Error** | **P-value** |
| Height timing Z-score | SEP 2 | -0.2 | 0.02 | <0.0001 | -0.3 | 0.02 | <0.0001 |
|  | SEP 3 | 0.01 | 0.03 | 0.7 | -0.01 | 0.04 | 0.7 |
|  | SEP 4 | 0.1 | 0.02 | 0.0003 | 0.2 | 0.02 | <0.0001 |
|  | SEP 5 | 0.2 | 0.03 | <0.0001 | 0.4 | 0.04 | <0.0001 |
|  | DOB x SEP 1 | -0.1 | 0.01 | <0.0001 | -0.1 | 0.02 | <0.0001 |
|  | DOB x SEP 2 | -0.02 | 0.02 | 0.3 | 0.02 | 0.02 | 0.3 |
|  | DOB x SEP 3 | 0.1 | 0.04 | 0.04 | 0.2 | 0.04 | <0.0001 |
|  | DOB x SEP 4 | 0.04 | 0.02 | 0.07 | 0.01 | 0.03 | 0.7 |
|  | DOB x SEP 5 | -0.06 | 0.03 | 0.03 | -0.1 | 0.04 | 0.015 |
|  | R^2^ adjusted 0.02 |  |  |  | R^2^ adjusted 0.05 |  |  |
| Weight timing Z-score | SEP 2 | -0.2 | 0.02 | <0.0001 | -0.16 | 0.02 | <0.0001 |
|  | SEP 3 | 0.4 | 0.03 | <0.0001 | 0.4 | 0.03 | <0.0001 |
|  | SEP 4 | 0.6 | 0.02 | <0.0001 | 0.6 | 0.02 | <0.0001 |
|  | SEP 5 | 0.9 | 0.03 | <0.0001 | 0.9 | 0.03 | <0.0001 |
|  | DOB x SEP 1 | -0.1 | 0.01 | <0.0001 | -0.1 | 0.02 | <0.0001 |
|  | DOB x SEP 2 | -0.03 | 0.02 | 0.048 | 0.02 | 0.02 | 0.2 |
|  | DOB x SEP 3 | 0.1 | 0.04 | 0.011 | 0.2 | 0.04 | 0.00003 |
|  | DOB x SEP 4 | -0.03 | 0.02 | 0.1 | -0.1 | 0.02 | 0.001 |
|  | DOB x SEP 5 | -0.2 | 0.03 | <0.0001 | -0.1 | 0.03 | 0.00004 |
|  | R^2^ adjusted 0.23 |  |  |  | R^2^ adjusted 0.15 |  |  |

Supplementary Table 1. Socioeconomic position (SEP) group and decimal date of birth (DOB, in decades) regressions (regression coefficient B, standard error, and P-value) for males and females in height, weight, BMI and grip strength SITAR timing Z-scores with SEP 1 as the reference group.

| **Males** | | | | **Females** | | | |
| --- | --- | --- | --- | --- | --- | --- | --- |
| **Dependent Variable** | **Predictor** | **B** | **Std. Error** | **P-value** | **B** | **Std. Error** | **P-value** |
| Height intensity Z-score | SEP 2 | -0.3 | 0.02 | <0.0001 | -0.4 | 0.02 | <0.0001 |
|  | SEP 3 | -0.7 | 0.03 | <0.0001 | -0.6 | 0.04 | <0.0001 |
|  | SEP 4 | -0.9 | 0.02 | <0.0001 | -0.7 | 0.02 | <0.0001 |
|  | SEP 5 | -1.2 | 0.03 | <0.0001 | -1.0 | 0.04 | <0.0001 |
|  | DOB x SEP 1 | -0.01 | 0.01 | 0.4 | -0.06 | 0.02 | 0.0007 |
|  | DOB x SEP 2 | 0.05 | 0.02 | 0.0007 | 0.06 | 0.02 | 0.003 |
|  | DOB x SEP 3 | 0.01 | 0.03 | 0.7 | 0.004 | 0.04 | 0.9 |
|  | DOB x SEP 4 | 0.1 | 0.02 | <0.0001 | 0.1 | 0.02 | 0.0005 |
|  | DOB x SEP 5 | 0.2 | 0.03 | <0.0001 | 0.2 | 0.03 | <0.0001 |
|  | R^2^ adjusted 0.14 |  |  |  | R^2^ adjusted 0.08 |  |  |
| Weight intensity Z-score | SEP 2 | 0.01 | 0.02 | 0.5 | 0.1 | 0.02 | <0.0001 |
|  | SEP 3 | -0.4 | 0.03 | <0.0001 | -0.02 | 0.04 | 0.5 |
|  | SEP 4 | -0.5 | 0.02 | <0.0001 | -0.1 | 0.02 | <0.0001 |
|  | SEP 5 | -0.8 | 0.03 | <0.0001 | -0.4 | 0.04 | <0.0001 |
|  | DOB x SEP 1 | 0.1 | 0.01 | <0.0001 | 0.1 | 0.01 | <0.0001 |
|  | DOB x SEP 2 | 0.04 | 0.01 | 0.04 | 0.05 | 0.02 | 0.03 |
|  | DOB x SEP 3 | -0.1 | 0.04 | 0.02 | -0.04 | 0.04 | 0.4 |
|  | DOB x SEP 4 | 0.05 | 0.02 | 0.02 | 0.05 | 0.02 | 0.04 |
|  | DOB x SEP 5 | 0.05 | 0.02 | 0.02 | 0.13 | 0.03 | 0.0003 |
|  | R^2^: adjusted 0.1 |  |  |  | R^2^: adjusted 0.03 |  |  |
| BMI intensity Z-score | SEP 2 | 0.2 | 0.01 | <0.0001 | 0.3 | 0.02 | <0.0001 |
|  | SEP 3 | -0.1 | 0.03 | 0.0007 | 0.3 | 0.04 | <0.0001 |
|  | SEP 4 | -0.1 | 0.02 | <0.0001 | 0.2 | 0.02 | <0.0001 |
|  | SEP 5 | -0.3 | 0.03 | <0.0001 | 0.3 | 0.04 | <0.0001 |
|  | DOB x SEP 1 | 0.1 | 0.01 | <0.0001 | 0.03 | 0.02 | 0.1 |
|  | DOB x SEP 2 | 0.1 | 0.02 | <0.0001 | 0.1 | 0.02 | <0.0001 |
|  | DOB x SEP 3 | -0.05 | 0.04 | 0.2 | -0.005 | 0.04 | 0.9 |
|  | DOB x SEP 4 | 0.1 | 0.02 | 0.00009 | 0.06 | 0.02 | 0.01 |
|  | DOB x SEP 5 | 0.1 | 0.03 | 0.002 | 0.04 | 0.03 | 0.26 |
|  | R^2^ adjusted 0.05 |  |  |  | R^2^ adjusted 0.02 |  |  |
| Grip intensity Z-score | SEP 2 | -0.2 | 0.02 | <0.0001 | -0.3 | 0.02 | <0.0001 |
|  | SEP 3 | -0.7 | 0.03 | <0.0001 | -0.6 | 0.03 | <0.0001 |
|  | SEP 4 | -0.9 | 0.02 | <0.0001 | -0.7 | 0.02 | <0.0001 |
|  | SEP 5 | -1.3 | 0.03 | <0.0001 | -1.2 | 0.03 | <0.0001 |
|  | DOB x SEP 1 | 0.02 | 0.01 | 0.15 | -0.02 | 0.02 | 0.3 |
|  | DOB x SEP 2 | 0.03 | 0.02 | 0.1 | 0.06 | 0.02 | 0.0004 |
|  | DOB x SEP 3 | -0.01 | 0.04 | 0.75 | -0.1 | 0.04 | 0.02 |
|  | DOB x SEP 4 | 0.1 | 0.02 | 0.00003 | 0.1 | 0.02 | <0.0001 |
|  | DOB x SEP 5 | 0.3 | 0.03 | <0.0001 | 0.3 | 0.03 | <0.0001 |
|  | R^2^ adjusted 0.17 |  |  |  | R^2^ adjusted 0.11 |  |  |

Supplementary Table 2. Socioeconomic position (SEP) group and decimal date of birth (DOB, in decades) regressions (regression coefficient B, standard error, and P-value) for males and females in height, weight, BMI and grip strength SITAR intensity Z-scores with SEP 1 as the reference group.

Supplementary Figure 1. Regression estimates of height and weight Z-scores with 95%CI (dot-whiskers) for timing across SEP groups (2-5), with SEP 1 as reference group (horizontal line at 0), by decade of birth centered on 1960, 1970, 1980 and 1990.

Supplementary Figure 2. Regression estimates of male height, weight, BMI and grip strength Z-scores with 95%CI (dot-whiskers) for intensity across SEP groups (2-5), with SEP 1 as reference group (horizontal line at 0), by decade of birth centered on 1960, 1970, 1980 and 1990.

Supplementary Figure 3. Regression estimates of female height, weight, BMI and grip strength Z-scores with 95%CI (dot-whiskers) for intensity across SEP groups (2-5), with SEP 1 as reference group (horizontal line at 0), by decade of birth centered on 1960, 1970, 1980 and 1990.

References for graphs:

Wickham H. ggplot2: Elegant Graphics for Data Analysis. New York: Springer-Verlag; 2016.

Robinson D, Hayes A. broom: Convert Statistical Analysis Objects into Tidy Tibbles. [Internet]. R package version 0.5.0.; 2018. Available from: <https://cran.r-project.org/package=broom>

Long J. _jtools: Analysis and Presentation of Social Scientific Data. [Internet]. R package version 1.1.0; 2018. Available from: <https://cran.r-project.org/package=jtools>

Stolt F, Hu Y. dotwhisker: Dot-and-Whisker Plots of Regression Results. [Internet]. R package version 0.5.0; 2018. Available from: <https://cran.r-project.org/package=dotwhisker>

Wickham H, Francois R, Henry L, Mueller K. dplyr: A Grammar of Data Manipulation. R package version 0.7.6.; 2018.
